# An Improved Deep Neural Network Model of Intelligent Vehicle Dynamics via Linear Decreasing Weight Particle Swarm and Invasive Weed Optimization Algorithms

**DOI:** 10.3390/s22134676

**Published:** 2022-06-21

**Authors:** Xiaobo Nie, Chuan Min, Yongjun Pan, Zhixiong Li, Grzegorz Królczyk

**Affiliations:** 1 College of Mechanical and Vehicle Engineering, Chongqing University, Chongqing 400044, China; xiaobo.nie@cqu.edu.cn (X.N.); chuan.min@cqu.edu.cn (C.M.); 2 State Key Laboratory of Structural Analysis for Industrial Equipment, Dalian University of Technology, Dalian 116024, China; 3 Faculty of Mechanical Engineering, Opole University of Technology, 45758 Opole, Poland; z.li@po.edu.pl (Z.L.); g.krolczyk@po.opole.pl (G.K.)

**Keywords:** longitudinal-lateral dynamics, vehicle multibody model, deep neural networks, particle swarm optimization, invasive weed optimization

## Abstract

We propose an improved DNN modeling method based on two optimization algorithms, namely the linear decreasing weight particle swarm optimization (LDWPSO) algorithm and invasive weed optimization (IWO) algorithm, for predicting vehicle’s longitudinal-lateral responses. The proposed improved method can restrain the solutions of weight matrices and bias matrices from falling into a local optimum while training the DNN model. First, dynamic simulations for a vehicle are performed based on an efficient semirecursive multibody model for real-time data acquisition. Next, the vehicle data are processed and used to train and test the improved DNN model. The vehicle responses, which are obtained from the LDWPSO-DNN and IWO-DNN models, are compared with the DNN and multibody results. The comparative results show that the LDWPSO-DNN and IWO-DNN models predict accurate longitudinal-lateral responses in real-time without falling into a local optimum. The improved DNN model based on optimization algorithms can be employed for real-time simulation and preview control in intelligent vehicles.

## 1. Introduction

The modeling of complex multibody system for vehicle dynamics and control is always a challenging task. First, the dynamic tire forces and vehicle-tire-road interactions are difficult to calculate, partially due to external disturbances and parameter perturbation [1,2,3]. Second, the multibody-based formulations lead to full-vehicle models with increased computational burden, which always makes the real-time simulation and control unavailable [4,5]. Furthermore, the numerical stability is also a challenging issue to consider for cases where an off-road vehicle passes through abnormal road surfaces [6,7,8]. Correspondingly, to overcome these issues, deep neural networks (DNNs) based on historical data are widely used for lateral dynamics and longitudinal-lateral dynamics modeling [9,10,11]. The DNN-based vehicle models are able to deal with complexity through automation. They are used to simulate various scenarios, collect a large amount of data, verify it in an automated simulation environment, and then test it on real vehicles. In this way, intelligent vehicles are constantly learning to deal with complex and realistic scenarios [12].

Theoretically, neural networks can approximate nonlinear continuous functions under the reasonable structure and appropriate weights. Hence, they are suitable for solving various inherent complex problems and can adapt to complex nonlinear mapping. Deep neural networks, on the other hand, can be regarded as a more complex form of neural networks. They possess many unmatched features compared to traditional statistical methods and algorithms, e.g., self-adaptation, self-organization, self-learning, high non-linearity, and good fault tolerance [13]. In one work, Rutherford et al. investigated the nonlinear modeling ability of neural networks to identify and control the dynamic systems [14]. In another related work, Kim et al. developed a scheme for sideslip angle estimation, which combines DNNs and Kalman filters for accurate prediction [15]. Similarly, Devineau et al. explored the capabilities of DNNs to perform lateral and longitudinal modeling of a vehicle [16]. Melzi et al. developed a layered neural-network method using dynamic parameters obtained from vehicle sensors to accurately estimate the sideslip angle [17]. Ji et al. developed a novel lateral motion control approach consisting of a robust steering controller and an adaptive neural network approximator [18]. Progressively, Acosta et al. used feed-forward neural networks and a model predictive controller for autonomous vehicle drifting along a large range of road radii [19]. Lio et al., in one recent work, investigated neural networks of different architectures for modeling longitudinal dynamics of a medium-scale vehicle [20].

Conclusively, the literature survey indicates that DNN-based approaches can be effectively used for vehicle dynamics modeling. The main difficulty for DNN methods, however, lies in the required number of dynamics maneuvers to build a representative dataset. Additionally, the direct estimation of vehicle characteristics is sometimes unavailable because numerous vehicle tests are needed for it. Vehicle dynamics maneuvers based on the commercial software, such as ADAMS and RecurDyn, are also time consuming [21,22,23]. To address this issue, data-driven modeling methods based on efficient data acquisition and DNNs provide an effective solution. Accordingly, in this study, a DNN-based dynamics model for vehicle’s longitudinal-lateral dynamics is presented. Furthermore, an efficient semirecursive multibody formulation, which performs real-time simulations for data acquisition, is used to capture the vehicle key characteristics.

Activation functions are used to determine nonlinear properties in DNNs. Generally, they are nonlinear functions, such as the tanh and sigmoid functions. The use of rectified linear function (ReLU) has increased recently. Moreover, to calculate and update the weight matrices of DNNs, a backpropagation approach is widely used as a de-facto standard algorithm for improved recognition performance while training DNNs [24]. However, the backpropagation approach frequently requires a longer time to converge. Besides, the solutions of weight matrices and bias matrices tend to fall into the local optimum. To address this issue, two optimization methods, called as linear decreasing weight particle swarm optimization (LDWPSO) and invasive weed optimization (IWO), are introduced in this study to calculate the weight and bias matrices. The introduced optimization algorithms can improve the training results of the DNNs with faster convergence.

The highlights of this work lie in three phases. First, an efficient semirecursive multibody dynamics formulation is implemented for a vehicle system to acquire training data. The semirecursive vehicle dynamics model can accurately take into account the nonlinearities of a vehicle system. Therefore, the vehicle’s longitudinal-lateral dynamics can be obtained. The data in this study are more accurate compared to the results obtained from decoupled or simplified models. Second, a DNN modeling method for vehicle’s longitudinal-lateral dynamics is developed based on the training data. Most importantly, LDWPSO and IWO algorithms are introduced to improve the robustness and accuracy of the DNN model [25,26,27]. Various applied torques and initial velocities, spanning over a large range, are used to imitate diverse driving situations (accelerating and decelerating). Lastly, the obtained numerical results are validated in details.

The rest of the work is organized as follows. In Section 2, we present an efficient semirecursive multibody formulation for real-time data acquisition. Furthermore, a vehicle dynamics modeling method via DNNs and obtained data is developed. In Section 3, we introduce LDWPSO and IWO algorithms relying on DNN vehicle model, to improve the training results. In Section 4, we analyze the effectiveness and accuracy of the proposed DNN model for different driving situations. Finally, in Section 5, we conclude our work.

## 2. DNN Modeling for Vehicle’s Longitudinal-Lateral Dynamics

### 2.1. Vehicle Dynamics Data Acquisition

In this study, we used an accurate and efficient semirecursive multibody formulation to model the vehicle dynamics and acquire the longitudinal-lateral characteristics. This semirecursive formulation was first proposed by García de Jalón et al. [28,29,30]. Based on this approach, the equations of motion for a vehicle multibody system can be concisely expressed as:

(1) $$R_{z}^{T}R_{d}^{T}M^{\Sigma }R_{d}R_{z}{\ddot{z}}^{i}=R_{z}^{T}R_{d}^{T}\left[Q^{\Sigma }-T^{T}\bar{M}\frac{d\left(TR_{d}R_{z}\right)}{dt}{\dot{z}}^{i}\right]$$

where, $R_{d}$ represents the first velocity transformation matrix that can be used to express the Cartesian velocities and accelerations via relative velocities and accelerations. $R_{z}$ represents the second velocity transformation matrix, which can be utilized to describe relative velocities and accelerations via independent relative velocities and accelerations. T describes the path matrix of the system, and can express the recursive system tree-topology. $\bar{M}$ corresponds to generalized mass matrix of the system. $M^{\Sigma }$ and $Q^{\Sigma }$ refer to the accumulated generalized mass matrix and external forces, respectively. Lastly, ${\dot{z}}^{i}$ and ${\ddot{z}}^{i}$ denote the independent relative velocities and accelerations, respectively. See [30] for more details.

We can see that the motion expression (Equation (1)) is more complicated than other multibody formulations reported in [31]. However, this expression yields a small set of independent relative accelerations ${\ddot{z}}^{i}$, which in turn results in a higher computational efficiency. Therefore, the equations of motion (Equation (1)) can be used for real-time simulation in low-cost hardware [32,33,34]. On the other hand, to mitigate the issues related to unavailability of direct estimation of vehicle characteristics and high computational costs linked to commercial software based vehicle dynamics simulations, this efficient multibody formulation is introduced for vehicle simulation and data acquisition.

**Figure 1:**
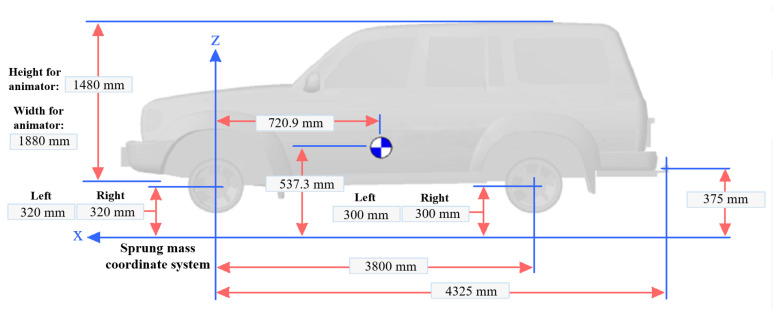
Vehicle system structure.

The vehicle system investigated in this study consists of Pacejka tire models, five-link suspensions in the rear, and McPherson suspensions in the front. Likewise, a schematic diagram of the vehicle multibody system is illustrated in Figure 1. Additional information related to the vehicle system is listed in Table 1. The vehicle multibody model has 34 relative (joint) coordinates in total, where 14 of them are independent of each other and can be utilized to describe the kinematics and dynamics of the full-vehicle model.

**Figure 2:**
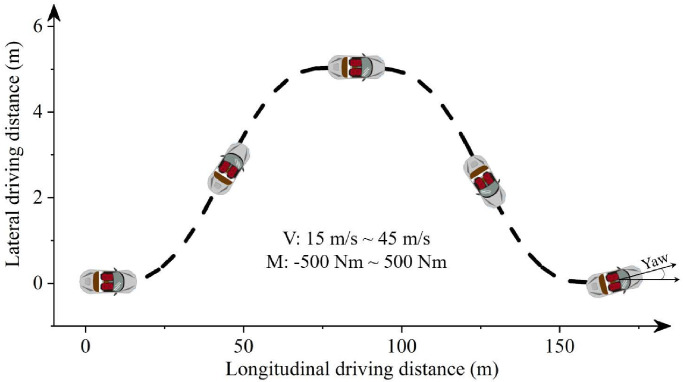
The driving track of the vehicle.

In the process of dynamic simulation and data acquisition, the vehicle is driven by various torques applied on front wheels and initial speeds. The driving torques range from −500 Nm to 500 Nm, to imitate the deceleration and acceleration conditions comprehensively. The initial speeds of the vehicle are set in a range spanning between 15 m/s and 45 m/s. The vehicle moves in a double lane change maneuver, as described in Figure 2. The vehicle simulation lasts for 5 s with time-step of 1 ms. Subsequently, 500 longitudinal-lateral dynamics datasets are collected. Each dataset includes vehicle initial speed, driving torque, longitudinal and lateral driving distances, final longitudinal and lateral velocities, and vehicle yaw angle.

The collected 500 datasets are randomly divided into the training and testing sets, containing 450 and 50 datasets, respectively. The training set is used to develop the DNN model, while the testing set is employed to evaluate the effectiveness of DNN model. In addition, all sample data is standardized during the data processing. Accordingly, the original data is transformed into non-dimensional index evaluation values via standardization. It prevents higher values from weakening the effects of lower values, which is essential to balance the contribution of each feature. Furthermore, standardization can also speed up the gradient descent to find optimal solutions. In this study, Z-score standardization was used for the data processing, which can be mathematically expressed as [35]:

(2) $$x^{*}=\frac{x-\mu }{\sigma }$$

where, *x* represents raw data of samples, μ represents the mean value of raw data, and σ represents the standard deviation of raw data. The term $x^{*}$ represents the processed data, which constitutes the input values for DNN model. After standardization, the standard deviation of processed data is 1 with an average value of 0. Additionally, reversed standardization is performed on the output values of DNN model.

### 2.2. DNN Model of the Vehicle

**Figure 3:**
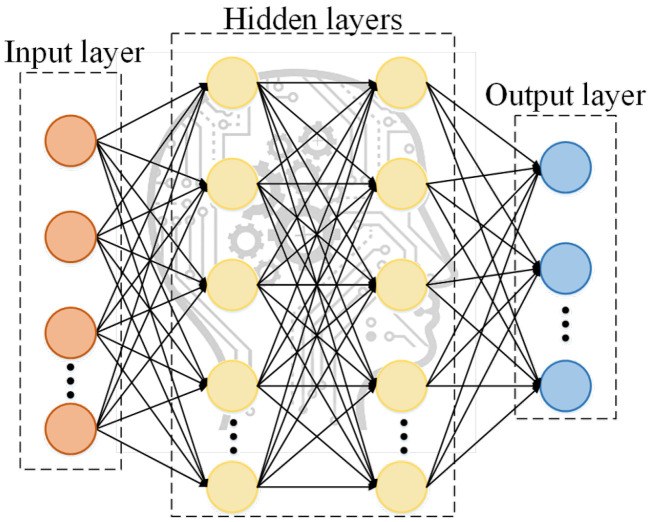
Deep neural network structure.

The principle of DNNs is briefly demonstrated in Figure 3. In DNN process, the relationship between training data is mapped to the output layer. By continuously adjusting the values of weight matrix and bias matrix, errors between the output results and expected values are reduced and controlled. As shown in Figure 3, the general DNNs mainly include an input layer, several hidden layers, and an output layer. Each neuron in one layer has a direct connection with a certain neuron in the next layer. The units of these networks use different activation functions for propagation in different applications, and there are no cycles or loops in the neural networks. In addition, the number of layers and the number of neurons in each layer are not limited. They require repeated adjustments to ensure that the accuracy requirements for predicted results are satisfied. An increase in number of layers and neurons corresponds to lower training efficiency of the neural networks. Besides, the elements of input layer neurons must be highly correlated with the elements predicted by an output layer. Moreover, they should be sensitive to changes in the predicted elements [36,37].

The learning process of DNNs involves forward propagation of the signal from an input layer and the backward propagation of error from the output layer. The input matrix, weight matrices, and bias matrices need to be defined in forward propagation. Accordingly, these matrices can be written as:

(3) $$Z_{1}=\left(i_{1},\;i_{2},\;i_{3},\;\cdots \;i_{m}\right)$$

(4) $$W_{n}=\left(w_{n_{1}},\;w_{n_{2}},\;w_{n_{3}},\;\cdots \;w_{n_{m}}\right)$$

(5) $$B_{n}=\left(b_{n_{1}},\;b_{n_{2}},\;b_{n_{3}},\;\cdots \;b_{n_{m}}\right)$$

where, $Z_{1}$ represents the input matrix of DNNs, $W_{n}$ represents *n*-th layer’s weight matrix, $B_{n}$ represents *n*-th layer’s bias matrix, *m* represents the number of samples in a training set, and *n* represents the total number of hidden and input layers. Progressively, the procedure for forward propagation can be expressed as:

(6) $$Z_{1}=A_{1}$$

(7) $$Z_{i+1}=W_{i}^{T}A_{i}+B_{i},\;i=1\ldots n$$

(8) $$A_{i+1}=f^{i+1}\left(Z_{i+1}\right),\;i=1\ldots n$$

where, $Z_{i}$ contains *i*-th layer’s input, $A_{i}$ contains *i*-th layer’s output, and $f^{i}$ represents the *i*-th activation function. Note that $A_{n+1}$ contains the output of the DNN model. In this study, neural networks consist of four layers, among which there are two hidden layers. The two hidden layers involve 28 and 15 neurons, respectively. The first two activation functions of DNNs are ReLU functions, while the last activation function is a linear function. The ReLU function is mathematically expressed as:

(9) f(x)=max(0,x)

For backward propagation, we must choose a suitable loss function to judge the errors between the results of DNN model and the results of the multibody model. By continuously adjusting the values of the weight matrices and bias matrices, value of loss function keeps decreasing during the process of backward propagation. Generally, with the decrease in value of loss function, the accuracy of the DNN model increases. In this study, the mean square error (MSE) was opted as a loss function, which can be expressed as:

(10) $$L=\frac{{∥Y-A_{n+1}∥}_{2}^{2}}{2m}$$

where, L denotes the loss of DNN model, Y contains the value of the samples, $A_{n+1}$ contains the results of DNN model, and *m* denotes the number of samples.

Traditionally, gradient descent is widely used for backward propagation in neural network training. Nevertheless, in recent years, the adaptive moment estimation (Adam) optimization algorithm has gained more attention in deep learning [38]. Likewise, Adam algorithm offers independent adaptive learning rates for different parameters by computing the first and second moment estimations of gradients [39]. It can be implemented easily and directly, thereby improving the computational efficiency of DNNs. The hyperparameters of Adam algorithm with intuitive interpretation usually do not require much tuning. Similarly, the Adam algorithm used in this study can be mathematically expressed as:

(11) $$m_{t}=\beta_{1}m_{t-1}+\left(1-\beta_{1}\right)dm_{t}$$

(12) $$n_{t}=\beta_{2}n_{t-1}+\left(1-\beta_{2}\right)dn_{t}$$

(13) $${\hat{m}}_{t}=\frac{m_{t}}{1-\beta_{1}^{t}}$$

(14) $${\hat{n}}_{t}=\frac{n_{t}}{1-\beta_{2}^{t}}$$

(15) $$\Delta \theta_{t}=-\frac{{\hat{m}}_{t}}{\sqrt{{\hat{n}}_{t}}+\varepsilon }\alpha$$

where, $m_{t}$ and $n_{t}$ represent the first and second moments of weight and bias matrices for *t*-th training iteration, respectively. The term ${\hat{m}}_{t}$ and ${\hat{n}}_{t}$ denote the corrected value of first and second moments for *t*-th training iteration, respectively. $\Delta \theta_{t}$ represents the corrected value of weight and bias matrices for *t*-th training iteration. $\beta_{1}$ and $\beta_{2}$, respectively, denote the attenuation coefficients of first and second moments, whose values generally are 0.9 and 0.999, respectively. Moreover, α can be regarded as a learning rate for gradient descent, and ε is a smoothing parameter with a value of 10 × 10^−8^, in this study.

**Figure 4:**
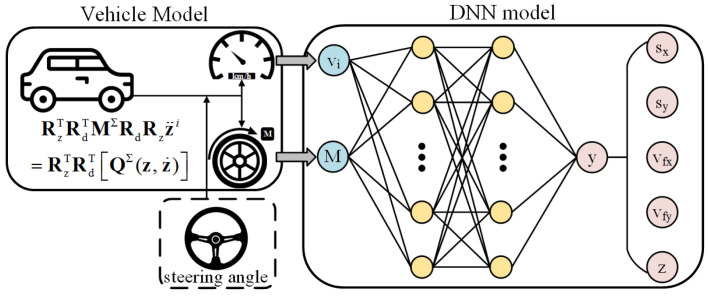
DNN model for vehicle’s longitudinal-lateral dynamics.

Additionally, the minibatch gradient descent is used to replace the stochastic gradient descent in backward propagation. In contrast to the stochastic gradient descent method, minibatch method randomly divides the entire dataset into a number of smaller batches, and performs Adam optimization according to the training results of each batch. Although its computational efficiency is relatively low, its training convergence is better than the stochastic gradient descent, in this way randomness of Adam optimization is reduced. Following the development of forward and backward propagation, a DNN model to predict the vehicle’s longitudinal-lateral dynamics is estimated. The DNN modeling procedure is described in Figure 4. However, in the process of updating the DNN model, the solutions of weight and bias matrices tend to fall into a local optimum [40]. Hence, to solve this issue, we introduce LDWPSO and IWO algorithms to improve the training results of the neural networks in the next section.

## 3. Optimization Algorithms

### 3.1. Linear Decreasing Weight Particle Swarm Optimization

The key advantages linked to particle swarm optimization (PSO) are its simple structure and rapid convergence. It has been widely used for applications in neural network training, kinetic modeling, multimodal function optimization, and control system [41,42]. In such an algorithm, a particle represents an individual and corresponds to a set of solutions, noting that particles have no mass and have only two attributes, namely position and speed of each particle. In each iteration, every particle of PSO explores for an optimal solution individually in a search space. Particles share information among each other to find the best individual value, which can be regarded as a current global optimal solution. Next, each particle corrects its position and speed according to the current global optimal solution. Thus, the PSO can effectively improve the performance of DNNs, and avoid its shortcomings of easily falling into local optimal values and network instability.

**Figure 5:**
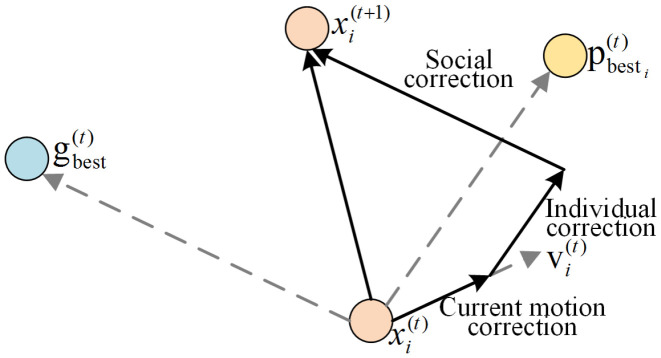
The procedure of particle position updating in PSO.

The updating equations of PSO can be described as follows. Note that, in this study, the objective function of PSO algorithm was used as a loss function of the neural networks.

(16) $$v_{i}^{\left(t+1\right)}=\omega v_{i}^{\left(t\right)}+c_{1}r_{1}\left({\mathrm{pbest}}_{i}^{\left(t\right)}-x_{i}^{\left(t\right)}\right)+c_{2}r_{2}\left({\mathrm{gbest}}_{i}^{\left(t\right)}-x_{i}^{\left(t\right)}\right)$$

(17) $$x_{i}^{\left(t+1\right)}=x_{i}^{\left(t\right)}+v_{i}^{\left(t+1\right)}$$

where, $x_{i}^{\left(t\right)}$ and $v_{i}^{\left(t\right)}$, respectively, denote the position and speed of *i*-th particle in *t*-th iteration. In this study, the dimensions of $x_{i}$ and $v_{i}$ are equal to the number of elements in $W_{n}$ and $B_{n}$, respectively. ${\mathrm{pbest}}_{i}^{\left(t\right)}$ and ${\mathrm{gbest}}_{i}^{\left(t\right)}$, respectively, denote the best position of *i*-th particle and the particle swarm in *t*-th iteration. $c_{1}$ and $c_{2}$ denote the corresponding acceleration factors, with values between 0 and 4. $r_{1}$ and $r_{2}$ denote the two random coefficients distributed from 0 to 1. Lastly, ω refers to the inertia factor and its value is non-negative. The particle position is updated as shown in Figure 5.

Noticeably, the value of inertia factor affects the optimization ability of PSO algorithm. Accordingly, a large value of inertia factor refers to a stronger global optimization ability, but weakens the local optimization ability simultaneously. In this study, the linear decreasing weight (LDW) method is utilized, i.e., the inertia factor decreases linearly with iterations. The LDW method is mathematically expressed as:

(18) $$\omega^{\left(t\right)}=\left(\omega_{ini}-\omega_{end}\right)\frac{\left(T-t\right)}{T}+\omega_{end}$$

where, $\omega_{ini}$ and $\omega_{end}$ denote the initial inertia factor and the end inertia weight, respectively. *T* represents the maximum number of iterations. The LDW equation ensures that PSO algorithm has better global search ability at the beginning of an iteration, and has more local search ability near the end of iteration.

**Figure 6:**
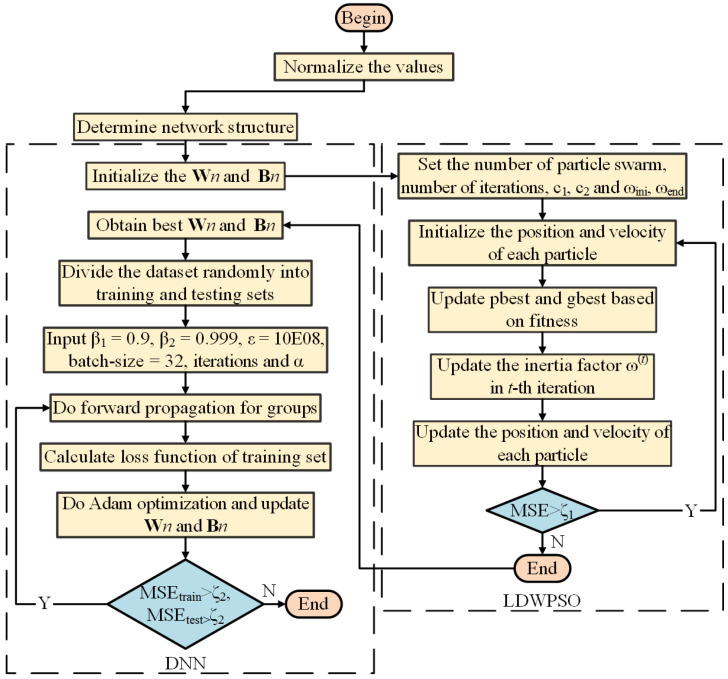
The framework of LDWPSO-DNN training.

**Figure 7:**
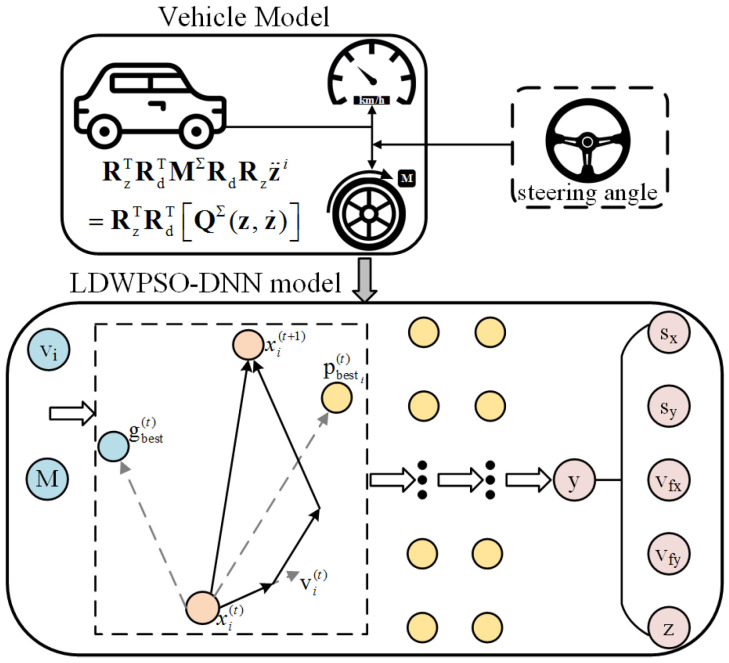
LDWPSO-DNN model for vehicle’s longitudinal-lateral dynamics.

The algorithm framework of the LDWPSO-DNN model is illustrated in Figure 6, while the LDWPSO-DNN modeling procedure is shown in Figure 7. Note that the input of LDWPSO is the acquired vehicle states, as mentioned above.

### 3.2. Invasive Weed Optimization

Invasive weed optimization (IWO) is a population-based numerical optimization algorithm. It corresponds to a meta-heuristic algorithm designed by simulating the colonial behavior of invasive weeds [43]. Contrary to other evolutionary algorithms, every weed reproduces an offspring during the process of evolution in IWO. Likewise, individuals with higher fitness produce more new individuals. Therefore, this algorithm strengthens the local search around superior individuals, while considering the diversity of the population. Owing to its strong robustness and adaptability, the IWO algorithm is widely used to solve practical engineering problems.

The IWO algorithm works in the following four phases. The first phase is population initialization, where a set of initial solutions is randomly generated in the D-dimensional search space. The number of initial solutions equals the initial population number. Note that, in this study, size of D is equal to the total number of elements in $W_{n}$ and $B_{n}$. Accordingly, the *i*-th initial solution can be written as:

(19) $$x_{i}=\left(x_{i1},x_{i2},x_{i3},...,x_{iD}\right)$$

Subsequently, the second phase refers to reproduction. In this phase, we calculate the fitness of each individual according to the defined objective function. Note that, in this study, objective function is the reciprocal of a loss function. The number of seeds that each individual can produce varies from the minimum to maximum based on the fitness value. The number of seeds reproduced by each weed can be expressed as follows:

(20) $$s_{i}=F_{floor}\left[s_{min}+\frac{f_{i}-f_{min}}{f_{max}-f_{min}}\left(s_{max}-s_{min}\right)\right]$$

where, $s_{i}$ represents the number of seeds reproduced by *i*-th weed, $F_{floor}$ denotes the round down function, $f_{i}$ denotes the fitness of *i*-th weed, $s_{max}$ and $s_{min}$ represent the maximum and minimum number of seeds that can be produced, respectively. $f_{max}$ and $f_{min}$ denote the maximum and minimum fitness values in the evolution of current generation, respectively.

The third phase of IWO algorithm is spatial dispersal. During this phase, the reproduced seeds are randomly distributed in D-dimensional search space, near the parent weeds, with a normal distribution. The mean value of the normal distribution is 0, and the standard deviation is $\delta_{t}$. The position of *s*-th seed produced by *i*-th weed is given as follows:

(21) $$x_{i,s}=x_{i}+N\left(0,\delta_{t}\right),\;\;\;s_{min}\leq s\leq s_{max}$$

where, $x_{i,s}$ denotes the position of *s*-th seed produced by the *i*-th weed. $\delta_{t}$ denotes the standard deviation of *t*-th iteration. The term $\delta_{t}$ can be calculated as follows:

(22) $$\delta_{t}=\left(\delta_{ini}-\delta_{end}\right){\left(\frac{T-t}{T}\right)}^{n}+\delta_{end}$$

where, $\delta_{ini}$ and $\delta_{end}$ denote the initial and end standard deviation, respectively. *T* represents the maximum number of iterations, and *n* is the non-linear adjustment factor, which equals 3 in this study.

**Figure 8:**
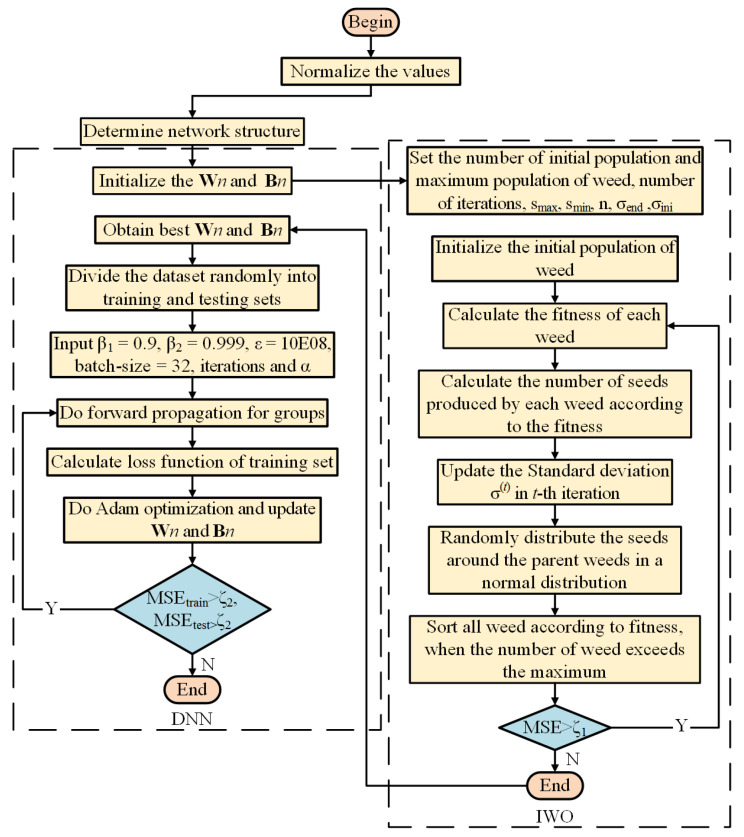
The framework of IWO-DNN training.

**Figure 9:**
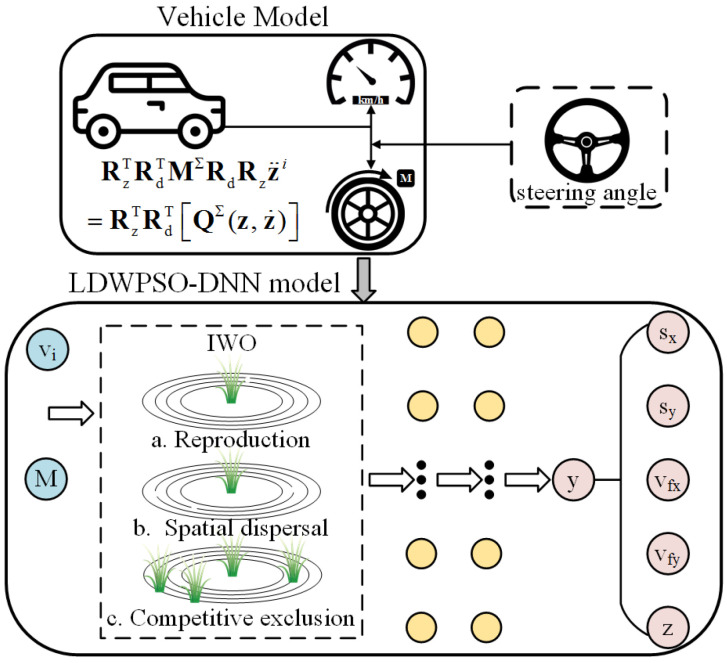
IWO-DNN model for vehicle’s longitudinal-lateral dynamics.

The fourth and last phase of algorithm is competitive exclusion. When a population size reaches its maximum, we sort all individuals according to the fitness value, exclude individuals with poor fitness, and keep the rest, which continue to evolve. The relevant framework and modeling procedure for IWO-DNN algorithm are elaborated in Figure 8 and Figure 9, respectively. Similar to the LDWPSO algorithm, input of IWO is the obtained vehicle states.

## 4. Numerical Results and Errors

### 4.1. Numerical Results

**Figure 10:**
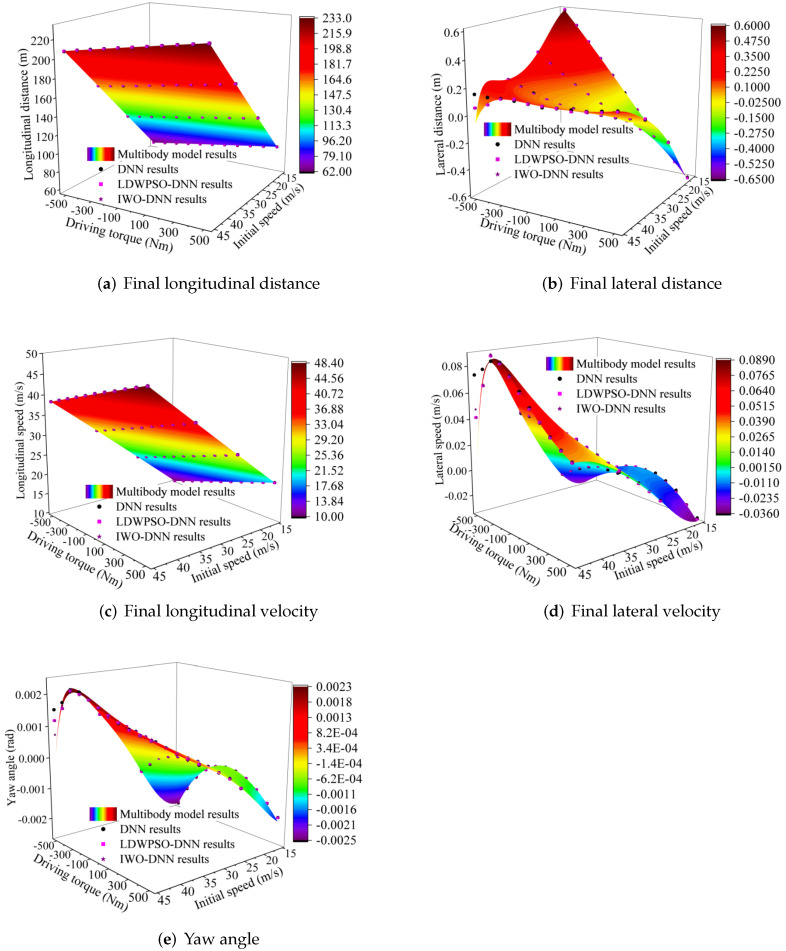
The comparison of DNN results.

To investigate the improvements, obtained through LDWPSO and IWO, in the training results of DNNs, the DNN outputs including longitudinal and lateral driving distances, final longitudinal and lateral velocities, and vehicle yaw angle are calculated and compared (given in Figure 10). Each figure involves multibody model results, DNN predicted results, and improved DNN predicted results. The driving situations, i.e., initial longitudinal speed varying from 15 m/s to 45 m/s and the driving torque ranging from −500 Nm to 500 Nm, were used to imitate the accelerating and decelerating.

**Figure 11:**
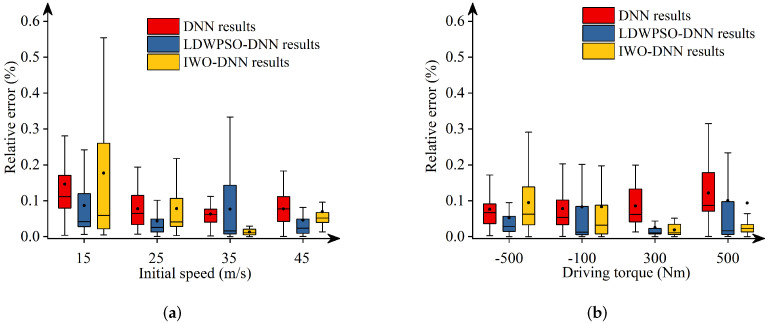
Absolute percentage error: final longitudinal distance.

**Figure 12:**
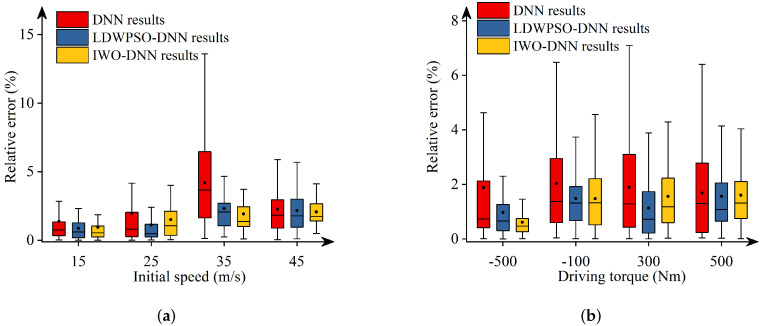
Absolute percentage error: final lateral distance.

**Figure 13:**
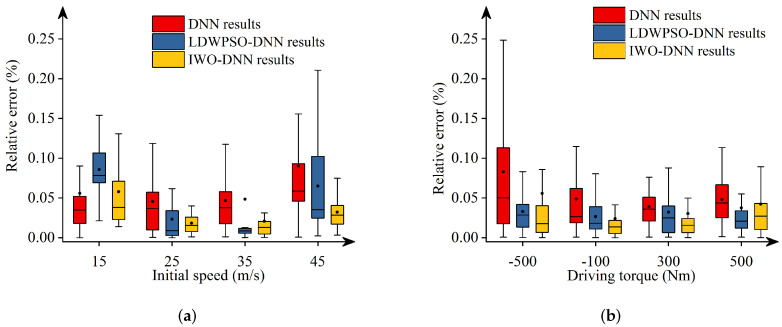
Absolute percentage error: final longitudinal velocities.

**Figure 14:**
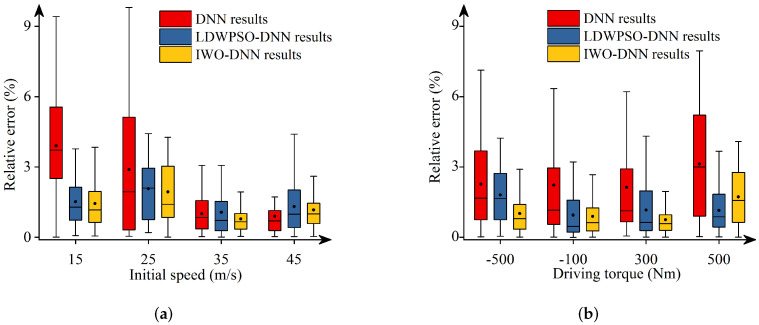
Absolute percentage error: final lateral velocities.

**Figure 15:**
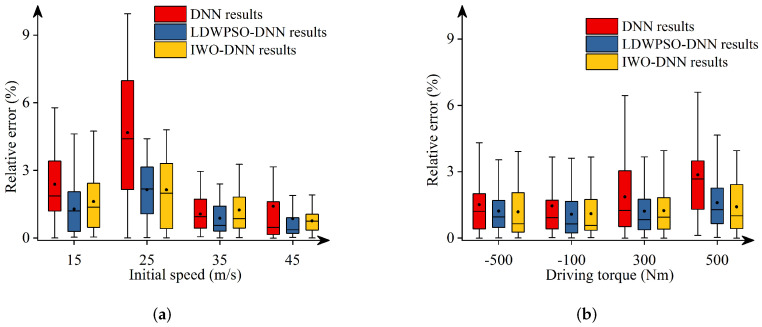
Absolute percentage error: yaw angle.

Additionally, Figure 11, Figure 12, Figure 13, Figure 14 and Figure 15 depict the absolute percentage errors for the above reported results in terms of five vehicle responses. Note that different initial longitudinal speeds are considered in these figures.

By analyzing the above numerical results, we can draw conclusions as follows:

- The predicted results of DNNs, LDWPSO-DNNs, and IWO-DNNs for longitudinal responses can fit the results of the vehicle multibody model well. The absolute percentage errors of less than 1% are observed.
- The absolute percentage errors of LDWPSO-DNNs and IWO-DNNs for lateral and longitudinal-lateral responses are smaller than that of DNNs. The results verify that LDWPSO and IWO algorithms improve the prediction accuracy of the DNN model.

### 4.2. Error Analysis

To quantify the improvements of LDWPSO and IWO algorithms to the accuracy of DNN results, we introduce four error functions, and compare the pros and cons for the predicted results. These four error functions are mean absolute error (MAE), mean absolute percentage error (MAPE), root mean square error (RMSE), and R^2^. The function MAE represents the average of absolute errors, and directly reflects the actual error of predicted results. The second one, MAPE is one of the widely used metrics for evaluating predictive performance, and can be calculated based on MAE easily. RMSE corresponds to arithmetic square root of MSE, which is more intuitive. For these three error functions, the smaller the value is, the better the predicted results are. Lastly, R^2^ denotes the coefficient of determination, with a range varying from 0 to 1. The model fits well if the value of R^2^ is closer to 1. Correspondingly, these four error functions are mathematically expressed as:

(23) $$\mathrm{MAE}=\frac{1}{n}\sum_{i=1}^{n}\left|y_{i}-{\hat{y}}_{i}\right|$$

(24) $$\mathrm{MAPE}=\frac{1}{n}\sum_{i=1}^{n}\left|\frac{y_{i}-{\hat{y}}_{i}}{y_{i}}\right|$$

(25) $$\mathrm{RMSE}=\sqrt{\frac{1}{n}{\sum_{i=1}^{n}\left(y_{i}-{\hat{y}}_{i}\right)}^{2}}$$

(26) $$R^{2}=1-\frac{{\sum_{i=1}^{n}\left(y_{i}-{\hat{y}}_{i}\right)}^{2}}{{\sum_{i=1}^{n}\left(y_{i}-{\bar{y}}_{i}\right)}^{2}}$$

where, $y_{i}$ represents the *i*-th value of reference results, ${\hat{y}}_{i}$ represents the *i*-th value of the predicted results, ${\bar{y}}_{i}$ represents the *i*-th mean value of the predicted results, and *n* represents the number of predicted results, which is 7701 for this study. Table 2, Table 3, Table 4, Table 5 and Table 6, respectively, depict evaluation results of final longitudinal and lateral distances, final longitudinal and lateral velocities, and yaw angle.

It is convenient to observe from Table 2, Table 3, Table 4, Table 5 and Table 6 that MAE, MAPE, and RMSE for LDWPSO-DNN and IWO-DNN models are smaller than the DNN model. The R^2^ values for LDWPSO-DNN and IWO-DNN models are closer to 1. Furthermore, for the final lateral and longitudinal velocities and yaw angle, the MAPE of LDWPSO-DNN and IWO-DNN models drops sharply. While analyzing the error functions of LDWPSO-DNN and IWO-DNN models, we can observe that most of the indicators for IWO-DNN model are better than the LDWPSO-DNN model.

## 5. Conclusions

In this study, we proposed a DNN-based method to model the longitudinal-lateral dynamics of a vehicle. Two additional optimization algorithms, namely LDWPSO and IWO, were used to enhance the performance of DNNs. To verify the effectiveness of the LDWPSO-DNN and IWO-DNN models, a vehicle system was modeled and the predicted vehicle states of three DNNs were compared with the reference results in terms of error functions. The comparative results reveal that the prediction accuracy of IWO-DNN model is higher than that of the other two DNN models. Overall, an improved DNN model using LDWPSO and IWO algorithms was introduced to describe the vehicle’s longitudinal-lateral dynamics in real time. The proposed method can be used effectively for the real-time simulation and control of intelligent vehicles in complex driving conditions to enhance the vehicle safety. The scenario-based testing of autonomous vehicles by using sensor networks will be performed to validate the proposed method in the future.

## Figures and Tables

**Table 1:** Critical parameters of the vehicle model.

Parameter	Value
Vehicle mass	1155 kg
Wheelbase	2.8 m
Centroid height	0.5373 m
Tire rolling radius	0.4673 kg
Stiffness of front absorber	40,000 N/m
Stiffness of rear absorber	35,000 N/m
Damping of front absorber	1800 N/(m/s)
Damping of rear absorber	1800 N/(m/s)
Distance from centroid to front axle	0.7209 m
Distance from centroid to rear axle	2.0791 m

**Table 2:** The accuracy analysis of final longitudinal distance.

Final Longitudinal Distance	MAE (m)	MAPE (%)	RMSE (m)	$R^{2}$
DNN	0.107476	0.078521	0.135354	0.999990
LDWPSO-DNN	0.071606	0.049622	0.138596	0.999990
IWO-DNN	0.062153	0.048728	0.135021	0.999990

**Table 3:** The accuracy analysis of final lateral distance.

Final Lateral Distance	MAE (m)	MAPE (%)	RMSE (m)	$R^{2}$
DNN	0.002647	9.346083	0.006920	0.998541
LDWPSO-DNN	0.002158	4.741057	0.003724	0.999577
IWO-DNN	0.001912	3.201640	0.003467	0.999634

**Table 4:** The accuracy analysis of final longitudinal velocity.

Final Longitudinal Velocity	MAE (m/s)	MAPE (%)	RMSE (m/s)	$R^{2}$
DNN	0.013769	0.050521	0.018431	0.999996
LDWPSO-DNN	0.009383	0.032383	0.015797	0.999997
IWO-DNN	0.007688	0.025955	0.015518	0.999997

**Table 5:** The accuracy analysis of final lateral velocity.

Final Lateral Velocity	MAE (m/s)	MAPE (%)	RMSE (m/s)	$R^{2}$
DNN	0.000524	7.754304	0.001778	0.996591
LDWPSO-DNN	0.000341	4.861110	0.000791	0.999325
IWO-DNN	0.000290	3.255596	0.000848	0.999224

**Table 6:** The accuracy analysis of the yaw angle.

Yaw Angle	MAE (rad)	MAPE (%)	RMSE (rad)	$R^{2}$
DNN	0.000014	6.804836	0.000040	0.998480
LDWPSO-DNN	0.000010	3.879418	0.000026	0.999348
IWO-DNN	0.000008	3.176424	0.000016	0.999734

## Data Availability

Not applicable.
